# The Wages and Employment Conditions of Unskilled Workers

**Published:** 1920-07-17

**Authors:** 


### HOSPITALS AND THE INDUSTRIAL COURT.

### The Wages and Employment Conditions of Unskilled Workers.

A decision just arrived at by the Industrial Court in a matter submitted to it for arbitration by consent of certain hospitals cannot fail to be of considerable interest to all institutions. In December last the representatives of certain Metropolitan hospitals were asked to attend a conference at the Ministry of Labour in respect to the wages and hours of their unskilled manual employees. This conference was called at the instance of the Dock, Wharf, Riverside, and General Workers' Union and the National Union of General Workers, who claimed co-ordination of wages and working conditions in the Metropolitan district. The hospitals were asked by the Ministry if they would meet the Unions' representatives, which was agreed, and at that meeting the following resolution was passed:—

"The representatives of the hospitals present are prepared to recommend to their respective Committees the desirability of establishing a standard basis of wages, but, having regard to the variable financial circumstances of the different hospitals, are unable to agree on a figure which would be generally applicable at the moment. They would be willing that the whole claim of the Unions as stated in the Dock, Wharf, Riverside, and General Workers' Union should be submitted to arbitration by the Arbitration Court."

### Terms of Reference.

The claim of the Unions was for the following minimum terms and conditions for their members employed at the hospitals mentioned, viz.:—

1. A minimum weekly wage of £3 5s. for adult male workers weekly.

2. A minimum weekly wage of £1 15s. for female workers.

3. A forty-eight-hour week.

4. Time and a half for all overtime outside the normal working day.

5. Time and a half for all work performed on Sundays.

6. Two weeks' holiday each year to all employees of one or more years' service.

### Industrial Court's Decision.

As will be seen later, the decision concerns only those hospitals represented at the Court—namely, the London, St. Mary's, Royal Free, Great Northern Central, King's College, Metropolitan, and Royal Waterloo Hospitals. It is pointed out by the Court that, with four exceptions, the parties now concerned include the principal hospitals of the Metropolitan area.

**The decision of the Court, which is dated July 2, is as follows :—**

1. Adult male workers shall receive a minimum wage of £3 per week in all cases in which meals are not provided.
2. Female workers aged eighteen years and over shall receive a minimum wage of £1 15s. per week for full-time workers in all cases in which meals are not provided.

In cases in which meals form part of the remuneration given the above minima shall be reduced by such an amount as may be agreed between the parties according to the circumstances of each individual case, based on the cost to the hospital.

3. A forty-eight-hours' week.

4. Time and a quarter for all time worked in excess of forty-eight hours in any one week.

5. The Court approves the agreement regarding Sunday work. [See below.]

6. Two weeks' holiday each year to all employees of one or more years' service.

### The Court's Composition and Proceedings.

The Industrial Court met on May 20. It consisted of four representatives of the Ministry of Labour, three of the Dock, Wharf, Riverside, and General Workers' Union, five of the National Union of General Workers, and representatives of the seven hospitals mentioned above. The Chairman was Mr. J. H. McLeod, C.B., of the Ministry of Labour; Mr. F. Thompson stated the case for the Unions, and Mr. Thomas Ryan was the spokesman for the hospitals. It was stated by the Union representative during the course of the proceedings that as originally intended the application was to cover far more hospitals than were represented, but Mr. Ryan made it clear at the outset that no other hospitals than those represented could be bound by any decision arrived at, and this was upheld by the Chairman. The classes of male workers concerned were indicated by Mr. Thompson to be in general porters, theatre staffs, laboratory attendants, men engaged as stokers, carriers, and such as those. There were other grades, he thought, who, once the minimum wage was settled, would rise proportionately. The female workers comprised principally charwomen, wardmaids, and scrubbers. It was pointed out and admitted that the questions under consideration were outside the usual lines of industrial action on account of the peculiar circumstances, financial and otherwise, of the institutions concerned, Mr. Ryan emphasising that regard must be had to the fact that the institutions were not run for profit, that the work was often comparatively light, that the practice existed of granting pensions, in many cases of supplying uniforms, and that the employees were treated on humane and considerate lines which could not be characteristic of ordinary industrial employment. Notwithstanding this, the hospitals agreed, as Mr. E. W. Morris later pointed out, that although a hospital is a charity, a charity is not to be run on the charity of its employees. A hospital should not go to generosity in wages, but it should to dead fair wages, and nothing beyond.

Details concerning rates of pay and hours of work at various hospitals were fully gone into, together with the special conditions inseparable from institution work and the varying arrangements in each. Mr. Ryan acknowledged the moderate and temperate spirit in which the case was put by the Unions, especially in the general statement. If the demand and some of the statement of detail supporting it had been quite as moderate the hospitals, he said, would not have had any complaint to make. On behalf of the hospitals, he intimated their willingness to concede the principle of a forty-eight-hours' week and a two weeks' holiday yearly after one year's service. They offered overtime beyond a forty-eight-hours' week at time and a quarter, and minimum wages of £2 15s. for adult males and £1 10s. for females.

### Sunday Labour.

The question of Sunday work, Mr. Ryan urged, could not be considered from the usual business standpoint. The hospitals, whilst willing to arrange that every worker should have a weekly break corresponding to the ordinary worker's Sunday, put strongly forward their claim that Sunday should rank equally with other days of the week. After consultation at the Court the Unions agreed to this on the understanding that a continuous break should be given every worker during the week equivalent to that given in institutions and businesses on which Sunday is now a normal working day. This agreement has been incorporated in the decision of July 2.

### Some Interesting Points.

During the proceedings it emerged that the fortnight's annual holiday is a practice at many hospitals already; that St. Bartholomew's and St. Thomas's Hospitals have had a forty-eight-hours' working week for some time, with payment for overtime at time-and-a-quarter, increasing in the case of St. Bartholomew's to time-and-a-half; that the weekly value of the food supplied to the men workers at the Great Northern Central Hospital, as computed by themselves, is 23s. per week; and that at St. Mary's Hospital the minimum wages have increased 139 per cent. since 1914.

**Hospitals and the Industrial Court**—(*continued*).

Some interesting figures were given by Mr. Ryan in relation to this last point. Since 1914 the cost of provisions had increased 97 per cent., that of surgery and dispensary, medicines, drugs, and dressings, 72 per cent., and of domestic expenses, including coal, fuel, washing, etc., 82 per cent. These results had been obtained by using less expensive articles, medicines, provisions, and a more rigid economy in quantities. Their system of buying now was not by contract, because they could not get contracts; they had to buy at the best prices they could get, which compared very closely with the retail prices which individuals had to pay. These figures should be borne in mind in considering the Board of Trade figure of 141 per cent. as the increased cost of living. This is based on the assumption that the same quantity and quality is bought now as before the war, whereas adjustments in these respects have had to be made by institutions as they have by individuals.

